# Relationship between qualitative and quantitative parameters of three-dimensional computed tomography, EGFR gene mutation, and ALK gene rearrangement in GGO-associated lung adenocarcinoma and their prognostic value

**DOI:** 10.3389/pore.2025.1612081

**Published:** 2025-09-11

**Authors:** Xinyan Liu, Yi Ren, Zihang Fei, Qingmei Shi, Li Lu

**Affiliations:** Chongqing Universal Hongling Medical Imaging Diagnostic Center, Chongqing, China

**Keywords:** three-dimensional CT, ground-glass opacities, lung adenocarcinoma, EGFR, ALK

## Abstract

**Objective:**

We aimed to analyze the relationship between the quantitative and qualitative parameters of three-dimensional computed tomography (CT), epidermal growth factor receptor (EGFR), and anaplastic lymphoma kinase (ALK) in ground-glass opacity (GGO)-associated lung adenocarcinoma and determine their prognostic value.

**Methods:**

In total, 208 patients with GGO-associated lung adenocarcinoma admitted to our hospital from January 2019 to September 2021 were selected as study subjects. All participants underwent EGFR gene mutation and ALK gene rearrangement tests. The quantitative and qualitative parameters of three-dimensional CT scans were compared among patients with different EGFR gene mutations and ALK gene rearrangements. Multivariate analysis was conducted to investigate the association of these parameters with EGFR gene mutation and ALK gene rearrangement in patients with GGO-associated lung adenocarcinoma. Furthermore, the quantitative and qualitative parameters of three-dimensional CT scans were compared among patients with different prognoses, and the value of these parameters in predicting patients’ prognoses was analyzed.

**Results:**

There were significant differences between patients with wild-type EGFR and patients with mutant EGFR in terms of the bronchial sign (BS), pleural indentation sign (PIS), vascular bundle sign (VBS), maximum nodule diameter (MND), nodule volume (NV), average CT value (ACTV), and solid compartment proportion (SCP) (*P* < 0.05). There were significant differences between patients with and without ALK gene rearrangement in terms of the BS, PIS, VBS, ACTV, and SCP (*P* < 0.05). There was a significant difference in BS, PIS, VBS, MND, NV, ACTV, and solidity between patients with favorable prognosis and those with poor prognosis (*P* < 0.05). The AUC of the combination of BS, PIS, VBS, MND, NV, ACTV, and SCP for predicting patients’ prognosis was the highest, significantly higher than the AUC value of individual parameters (*P* < 0.05).

**Conclusion:**

The quantitative and qualitative parameters of three-dimensional CT are closely associated with EGFR gene mutations, ALK gene rearrangements, and prognosis in patients with GGO-associated lung adenocarcinoma. Moreover, each parameter holds a high value in predicting the prognosis of patients with GGO-associated lung adenocarcinoma.

## Introduction

Based on recent reports, the incidence and mortality rates of lung cancer in China rank first among all malignant tumors, accounting for 22.0% of all new cancer cases and 28.5% of cancer-related deaths [1]. Lung adenocarcinoma is a common pathological type of lung cancer. Most patients with lung adenocarcinoma often have no obvious clinical symptoms in the early stages, and the cancer usually manifests as pulmonary nodules. With the increasing popularity of imaging technologies and growing awareness of physical examinations in recent years, the detection rate of ground-glass opacity (GGO)-associated lung adenocarcinoma has significantly increased [2]. GGO, an imaging manifestation on chest computed tomography (CT), appears as focal, cloudy, and hazy pale shadows with a higher density than normal lung tissue. CT is widely used in lung cancer screening, thereby improving the detection rate of GGO. Among lung nodules, there are many partial solid nodules with characteristics intermediate between those of pure ground-glass and solid nodules. Pure ground-glass and solid nodules may share the same imaging features, which can affect the interpretation of results [3]. With the continuous advancement of CT technology, three-dimensional CT scans can reconstruct three-dimensional stereoscopic lesions from multiple angles and directions, enabling the visualization of lesion characteristics in three dimensions and offering high value in the differential diagnosis of benign and malignant lung lesions, including GGO [4].

Epidermal growth factor receptor (EGFR) mutations and anaplastic lymphoma kinase (ALK) rearrangements are common genetic alterations in lung adenocarcinoma. Lung biopsy is an invasive procedure that may increase the risk of pneumothorax, hemorrhage, and vascular injury [5]. Previous studies have primarily explored the relationship between CT imaging features, EGFR mutations, and ALK rearrangements in patients with lung cancer, often focusing separately on qualitative or quantitative parameters [6, 7]. However, few studies have comprehensively analyzed both qualitative and quantitative parameters of three-dimensional CT in relation to these genetic alterations and evaluated their prognostic significance. Therefore, this study aimed to provide a reference for understanding the imaging characteristics of lung adenocarcinoma associated with GGO.

## Subjects and methods

### Subjects

In total, 208 patients with GGO-associated lung adenocarcinoma admitted to our hospital from January 2019 to September 2021 were included as study subjects, including 139 males and 69 females. They were aged 46–72 years (59.41 ± 6.35 years). The tumor was on the left side in 118 cases and on the right side in 90 cases. Regarding clinical stage, 83 patients were in stage I and 125 patients were in stage IIA. Regarding pathological types, there were 79 cases of invasive adenocarcinoma, 86 cases of minimally invasive adenocarcinoma, 27 cases of adenocarcinoma *in situ*, and 16 cases of atypical adenomatous hyperplasia.

The inclusion criteria were as follows: 1) lung adenocarcinoma meeting the diagnostic criteria of the Chinese Standard for the Diagnosis and Treatment of Primary Lung Cancer (2015 Edition) [8]; 2) GGO-associated lung adenocarcinoma; 3) patient undergoing EGFR and ALK gene rearrangement testing; and 4) good quality of three-dimensional CT images. The exclusion criteria were as follows: 1) coexistence of other malignant tumors and other types of lung cancer; 2) undergoing surgery, radiotherapy, or chemotherapy 3 months before enrollment; 3) history of previous lung surgery; 4) hematologic diseases; or 5) mental or intellectual disabilities.

### Methods

#### Three-dimensional CT examination

A Philips iCT 256-slice CT scanner was used for lung examination. The patients were in the supine position and instructed to hold their breath after deep inhalation. Scanning was conducted from the lung tip to the posterior costodiaphragmatic angle. Tube voltage, pitch, layer thickness, and matrix were 120 kV, 0.6 mm, 5 mm, and 512 × 512, respectively. For the lung window, we adopted a window width of 1500 Hu and a window position of 400 Hu. For the mediastinal window, the window width was 400 Hu and the window position was 40 Hu. The acquired two-dimensional images were transmitted to the workstation. The area of interest of the lesion was delineated, and the three-dimensional images were reconstructed. Two senior radiologists analyzed the three-dimensional images and obtained quantitative parameters, including the maximum nodule diameter (MND) and nodules volume (NV), average CT value (ACTV), and solid compartment proportion (SCP). Multi-plane reconstruction, maximum intensity projection, and other techniques were employed to observe the morphology of GGO. Qualitative indicators were obtained, including lesion location, nodule type, tumor shape, lobulation sign, cavitation, burr sign, vacuolar sign, bronchial sign (BS), pleural indentation sign (PIS), and vascular bundle sign (VBS).

#### Detection of EGFR and ALK gene rearrangement

**FIGURE 1:**
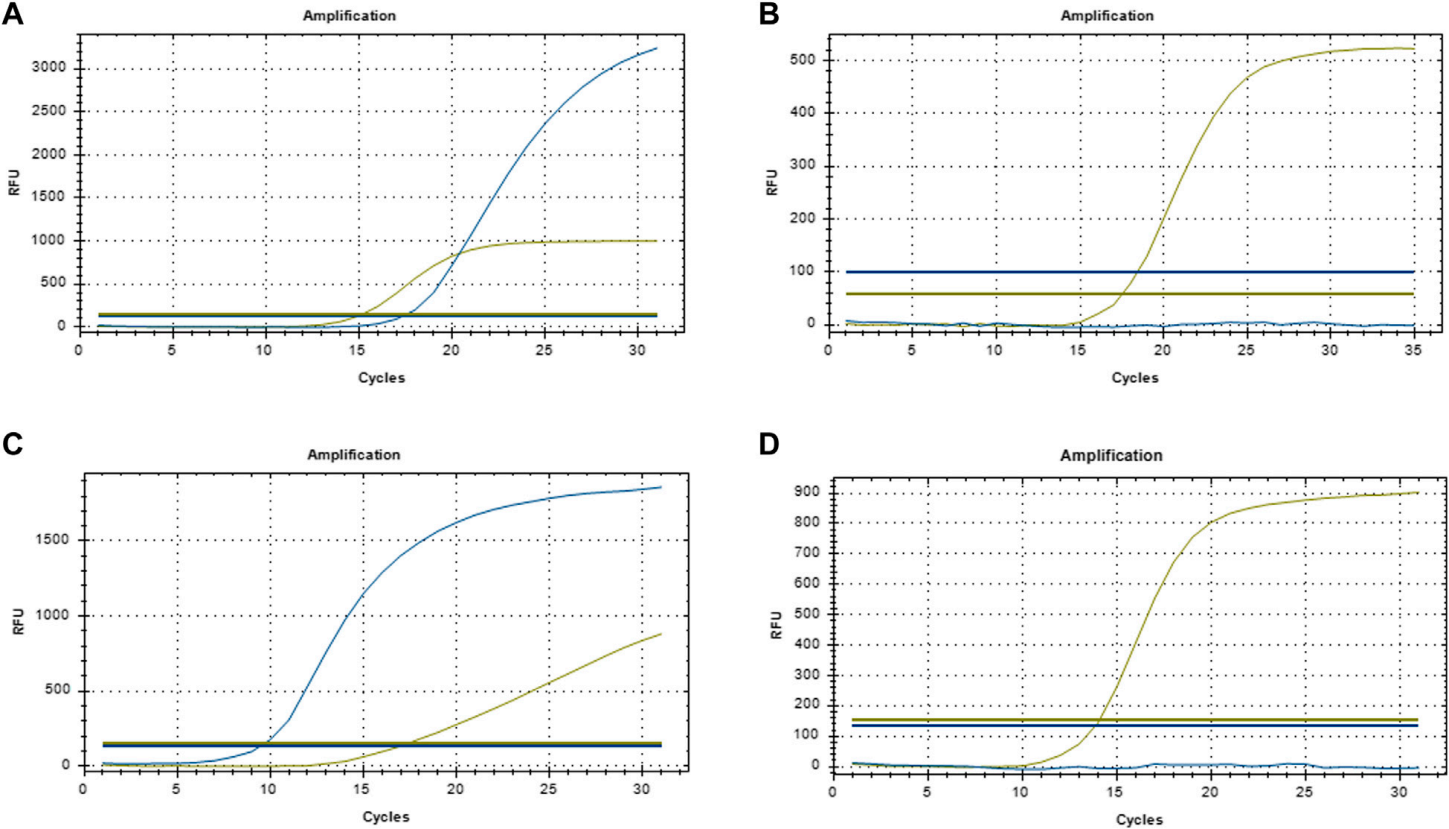
Representative qPCR amplification curve **(A)** EGFR-positive; **(B)** EGFR-negative; **(C)** ALK-positive; **(D)** ALK-negative.

Peripheral blood samples (4–5 mL) were collected from patients, and DNA was extracted using the QIAamp DNA Kit (Qiagen, Cat. No. 51183). DNA concentration was determined, and A260/280 was qualified in the range of 1.8∼2.0. The qualitative EGFR mutation detection kit and ALK fusion gene qualitative mutation detection kit (both from Xiamen AmoyDx Biotechnology Co., Ltd.; No. ADx-EG0X, ADx-AE01) were used following the manufacturers’ instructions to prepare the reaction system. PCR amplification was conducted using an ABI 7500 instrument under the following conditions: initial denaturation at 95°C for 5 min, followed by 15 cycles of 95°C for 25 s, 64°C for 20 s, and 72°C for 20 s; then 31 cycles of 93°C for 25 s, 60°C for 35 s, and 72°C for 20 s. EGFR mutations and ALK gene rearrangements were detected using the ARMS-PCR method. EGFR mutations were determined by comparing the sample Ct value with the threshold (ΔCt = sample Ct - quality control Ct). A Ct value <26 was regarded as a strong positive mutation. If 26≤Ct<29 and any of the following criteria were met the sample was classified as mutation-positive: ΔCt <11 for 19Del (exon 19), <11 for L858R (exon 21), <7 for T790M (exon 20), <9 for 20Ins (exon 20), <7 for G719X (exon 18), <8 for S768I (exon 20), or <8 for L861Q (exon 21). A Ct value of <26 was considered positive for ALK gene rearrangement. The primers used in this study were all designed and synthesized by the Beijing Genomics Institute (BGI). The representative qPCR amplification curves are shown in Figure 1. The primer sequences are shown in Table 1.

**TABLE 1:** Primer sequence.

Title	Primer
19Del	Forward: 5′-GCA​CCA​TCT​CAC​AAT​TGC​CAG​TTA-3′
	Reverse: 5′-ATG​TGG​AGA​TGA​GCA​GGG​TCT-3′
T790M	Forward: 5′-CCA​TGA​GTA​CGT​ATT​TTG​AAA​CTC-3′
	Reverse: 5′-CAT​ATC​CCC​ATG​GCA​AAC​TCT​TGC-3′
L858R	Forward: 5′-CCT​CAC​AGC​AGG​GTC​TTC​TCT​GT-3′
	Reverse: 5′-TCC​CTG​GTG​TCA​GGA​AAA​TGC​T-3′
20Ins	Forward: 5′-GAG​GCA​CCC​AGC​ACC​TTC​TT-3′
	Reverse: 5′-CAG​GGT​CTG​GAG​CAA​ATG​CT-3′
G719X	Forward: 5′-CTT​GTC​GCT​GGA​CAT​ACT​GG-3′
	Reverse: 5′-CCT​CCT​TAC​TTT​GCC​TCC​TTC​C-3′
S768I	Forward: 5′-GGA​CGT​ACT​GGT​GAA​AAC​ACC-3′
	Reverse: 5′-CAG​GGT​CTG​GAG​CAA​ATG​CT-3′
L861Q	Forward: 5′-CCT​CAC​AGC​AGG​GTC​TTC​TCT​GT-3′
	Reverse: 5′-TCC​CTG​GTG​TCA​GGA​AAA​TGC​T-3′
EML4-ALK	Forward: 5′-CCT​GAG​TCA​CAG​TGT​TTG​AGC-3′
	Reverse: 5′-TGC​CAG​CAA​AGC​AGT​AGT​TGG-3′

#### Prognostic assessment criteria

In total, 208 patients with GGO-associated lung adenocarcinoma were followed for 3 years, among whom nine were lost to follow-up and 199 completed follow-up. The last deadline for follow-up was September 2024.

### Outcome measures

(1) The qualitative and quantitative parameters of three-dimensional CT in patients with GGO-associated lung adenocarcinoma with/without EGFR and ALK gene rearrangement were compared. (2) Qualitative and quantitative parameters of three-dimensional CT associated with EGFR and ALK gene rearrangement in patients with GGO-associated lung adenocarcinoma were identified. (3) The association between the qualitative and quantitative parameters of three-dimensional CT and the prognosis of patients with GGO-associated lung adenocarcinoma was investigated. (4) The predictive value of qualitative and quantitative parameters of three-dimensional CT in the prognosis of GGO-associated lung adenocarcinoma was evaluated.

### Statistical analysis

The software SPSS 25.0 was used to analyze data. Measurement data with normal distribution and homogeneous variance are presented as $\bar{x}$ ± s and were compared between the two groups using an independent sample t-test. Measurement data without normal distribution are presented as median [M (Q1, Q2)] and were compared using the Mann-Whitney U test. Count data are presented as *n* (%) and were compared using the χ^2^ test. The receiver operating characteristic (ROC) curve and area under the curve (AUC) were employed to evaluate the prognostic value. A multicollinearity test revealed that the tolerance of meaningful quantitative and qualitative parameters in three-dimensional CT imaging of patients with EGFR and ALK mutations was >0.2, suggesting a low chance of multicollinearity. Multivariate logistic regression analysis was conducted with EGFR gene rearrangements as the dependent variable (wild-type = 1, mutant = 2) and BS (yes = 1, no = 0), PIS (yes = 1, no = 0), VBS (yes = 1, no = 0), MND (actual value), NV (actual value), ACTV (actual value), and SCP (actual value) as independent variables. *P* < 0.05 was considered statistically significant.

## Results

### Qualitative and quantitative parameters of three-dimensional CT in patients with GGO-associated lung adenocarcinoma and EGFR and ALK gene rearrangement

**FIGURE 2:**
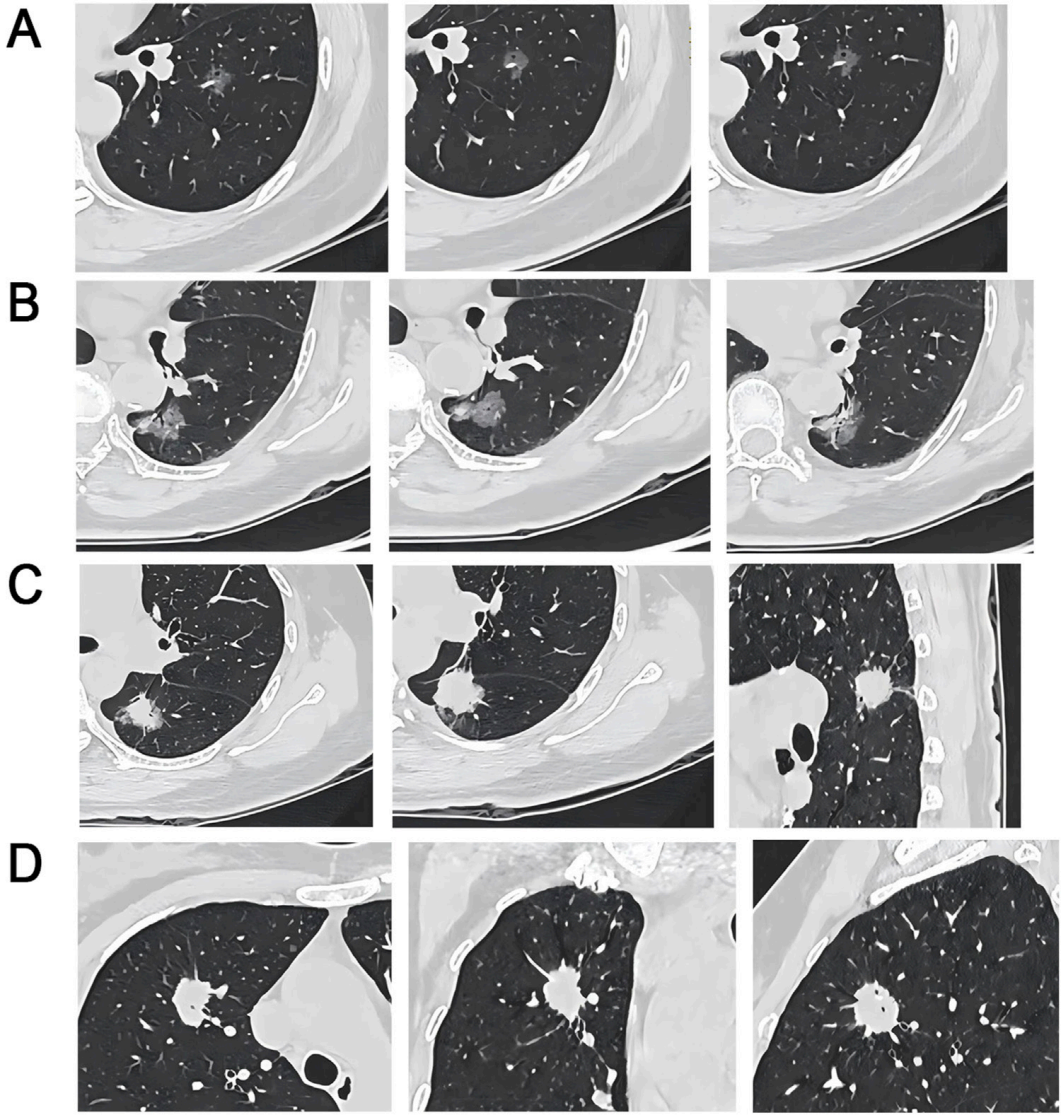
CT Imaging Characteristics of EGFR Wild-Type and ALK-Positive/Negative Lung Nodules **(A)** EGFR wild-type patient, 61-year-old female, pure GGO in the left lower lobe with vacuole, lobulation, spiculation, and PIS; **(B)** Circular EGFR wild-type, 75-year-old female, mixed GGO in the left lower lobe with pleural indentation, bronchial, vacuole, lobulation, and PIS; **(C)** ALK-negative, 56-year-old female, sub-solid nodule in the left lower lobe with bronchial, pleural indentation, spiculation, and deep lobulation signs; **(D)** ALK-positive, 51-year-old female, solid nodule in the right upper lobe with vacuole and spiculation signs.

Among 208 patients with GGO-associated lung adenocarcinoma, 121 (58.17%) had wild-type EGFR, while 87 (41.83%) had mutated EGFR. There were two cases with exon 18 mutations, 41 cases with exon 19 deletions, 10 cases with exon 20 insertions, and 34 cases with exon 21 L858R point mutations. There were 16 positive cases (7.69%) and 192 negative cases (92.31%) for the ALK gene rearrangement. There were significant differences between patients with wild-type EGFR and patients with mutant EGFR in terms of the BS, PIS, VBS, MND, NV, ACTV, and SCP (*P < 0.05*). There were significant differences between ALK gene rearrangement-positive and ALK gene rearrangement-negative patients in terms of the BS, PIS, VBS, ACTV, and SCP (*P < 0.05*) (Table 2). Four representative images were selected (Figure 2).

**TABLE 2:** Comparison of qualitative and quantitative parameters of three-dimensional CT in patients with different EGFR and ALK gene rearrangements [n(%)/( $\bar{x}$ ± s)].

Parameters	EGFR gene mutated		*t*/*χ* ^*2*^ */P*	ALK gene rearrangement		*t*/*χ* ^*2*^ */P*
	Wild-Type (121 cases)	Mutant-Type (87 cases)		Negative (192 cases)	Positive (16 cases)	
Qualitative parameters						
Location of the lesion			0.906/0.341			1.190/0.275
Left	72 (59.50)	46 (52.87)		111 (57.81)	7 (43.75)	
Right	49 (40.50)	41 (47.83)		81 (42.19)	9 (56.25)	
Type of nodule			1.032/0.310			0.060/0.807
Simplex	53 (43.80)	32 (36.78)		78 (40.63)	7 (43.75)	
Hybrid	68 (56.20)	55 (63.22)		114 (59.38)	9 (56.25)	
Tumor shape			0.878/0.349			2.258/0.133
Round/quasi-round	63 (52.07)	51 (58.62)		55 (28.65)	8 (50.00)	
Irregular	58 (47.93)	36 (41.38)		137 (71.35)	8 (50.00)	
Lobulation sign	92 (76.03)	61 (70.11)	0.911/0.340	141 (73.44)	12 (75.00)	0.025/0.874
Burr sign	64 (52.89)	57 (65.52)	3.315/0.069	110 (57.29)	11 (68.75)	0.797/0.372
Vacuolar sign	26 (21.49)	18 (20.69)	0.019/0.889	39 (20.31)	5 (31.25)	0.505/0.477
Empty	15 (12.440)	12 (13.79)	0.087/0.768	25 (13.02)	2 (12.50)	0.107/0.743
BS	30 (24.79)	39 (44.83)	9.163/0.002	57 (29.69)	12 (75.00)	13.679/<0.001
PIS	27 (22.31)	39 (44.83)	11.842/0.001	56 (29.17)	10 (62.50)	7.575/0.006
VBS	29 (23.97)	46 (52.87)	18.342/<0.001	65 (33.85)	11 (68.75)	7.756/0.005
Quantitative indicators						
MND (mm)	14.87 ± 4.89	18.83 ± 4.78	5.815/<0.001	15.24 (9.75,23.10)	16.72 (6.62,28.78)	2.826/0.078
NV(mm^3^)	485.78 ± 95.68	974.13 ± 268.71	18.445/<0.001	688.86 ± 173.49	704.20 ± 218.57	0.333/0.740
ACTV(HU)	−608.74 ± 236.78	−518.97 ± 165.18	3.043/0.003	−657.85 ± 158.96	−468.73 ± 125.68	4.636/<0.001
SCP(%)	12.68 ± 5.15	19.68 ± 5.67	9.268/<0.001	15.45 (6.95,32.42)	18.78 (5.13,35.69)	19.87 < 0.001

### The association between the qualitative and quantitative parameters of three-dimensional CT and EGFR and ALK gene rearrangement in GGO-associated lung adenocarcinoma

The multi-comparison multicollinearity test showed that the tolerance of meaningful quantitative and qualitative parameters in the three-dimensional CT of patients with EGFR and ALK mutations was >0.2, and the possibility of multicollinearity was small. The results indicated that BS, PIS, VBS, MND, NV, ACTV, and SCP were associated with EGFR mutation in GGO-associated lung adenocarcinoma (Table 3).

**TABLE 3:** Multivariate logistic regression results of EGFR gene rearrangements.

Influencing factors	*β*	*SE*	*Wald x* ^*2*^	*OR*	95%*CI*		*P*
					Lower limit	Upper limit	
BS	1.394	0.265	27.675	4.031	1.856	8.756	0.010
PIS	2.316	0.359	41.614	10.134	6.789	15.126	<0.001
VBS	1.598	0.424	14.233	4.942	2.246	10.873	0.006
MND	0.257	0.125	4.214	1.293	1.125	1.485	<0.001
NV	0.414	0.149	7.712	1.513	1.132	2.021	<0.001
ACTV	−1.313	0.258	25.900	0.269	0.158	0.458	<0.001
SCP	0.340	0.128	7.038	1.404	1.257	1.569	0.001

Multivariate logistic regression analysis was conducted using ALK gene rearrangement status as the dependent variable (negative = 1, positive = 2) and BS, PIS, VBS, ACTV, and SCP as independent variables. The results revealed that these factors were correlated with ALK gene rearrangement in GGO-related lung adenocarcinoma (Table 4).

**TABLE 4:** Multivariate logistic regression results of ALK gene rearrangement.

Influencing factors	*β*	*SE*	*Wald x* ^*2*^	*OR*	95%*CI*		*P*
					Lower limit	Upper limit	
BS	1.548	0.357	18.809	4.703	2.268	9.754	<0.001
PIS	1.921	0.478	16.144	6.825	3.453	13.489	<0.001
VBS	1.368	0.302	20.513	3.927	1.418	10.874	<0.001
ACTV	−0.736	0.159	21.400	0.479	0.268	0.857	<0.001
SCP	0.316	0.147	4.611	1.371	1.157	1.625	0.007

### Prognostic prediction in GGO-associated lung adenocarcinoma based on three-dimensional CT qualitative and quantitative parameters

In total, 208 patients were followed for 3 years, among whom nine were lost to follow-up. Among 199 patients with complete follow-up, 53 suffered from recurrence, metastasis, or death and were classified as having a poor prognosis, while the remaining 146 patients were classified as having a good prognosis. There were significant differences in BS, PIS, VBS, MND, NV, ACTV, and SCP between patients with good prognosis and those with poor prognosis (*P < 0.05*) (Table 5).

**TABLE 5:** Comparison of qualitative and quantitative parameters of three-dimensional CT in patients with differential prognosis [n(%)/( $\bar{x}$ ± s)].

Parameters	Favorable prognosis (146 cases)	Unfavorable prognosis (53 cases)	*t*/*χ* ^*2*^ */P*
Qualitative parameters			
Location of the lesion			1.598/0.206
Left	79 (54.11)	34 (64.15)	
Right	67 (45.89)	19 (35.85)	
Type of nodule			0.886/0.347
Simplex	58 (39.73)	25 (47.17)	
Hybrid	88 (60.27)	28 (52.83)	
Tumor shape			1.051/0.305
Round/quasi-round	79 (54.11)	33 (62.26)	
Irregular	67 (45.89)	20 (37.74)	
Lobulation sign	108 (73.97)	42 (79.25)	0.582/0.445
Burr sign	83 (56.82)	32 (60.38)	0.198/0.656
Vacuolar sign	25 (17.12)	13 (24.53)	1.380/0.240
Empty	17 (11.64)	8 (15.09)	0.421/0.516
BS	38 (26.03)	26 (49.06)	9.452/0.002
PIS	33 (22.60)	25 (47.17)	11.364/0.001
VBS	39 (26.71)	28 (52.83)	11.877/0.001
Quantitative indicators			
MND (mm)	13.285 ± 3.97	19.45 ± 3.69	9.862/<0.001
NV(mm^3^)	512.68 ± 102.37	933.18 ± 245.85	17.044/<0.001
ACTV(HU)	−626.87 ± 189.74	−478.21 ± 157.83	5.097/<0.001
SCP(%)	13.15 ± 4.02	17.67 ± 5.18	6.470/<0.001

### Predictive value of qualitative and quantitative parameters of three-dimensional CT for the prognosis of GGO-associated lung adenocarcinoma

**FIGURE 3:**
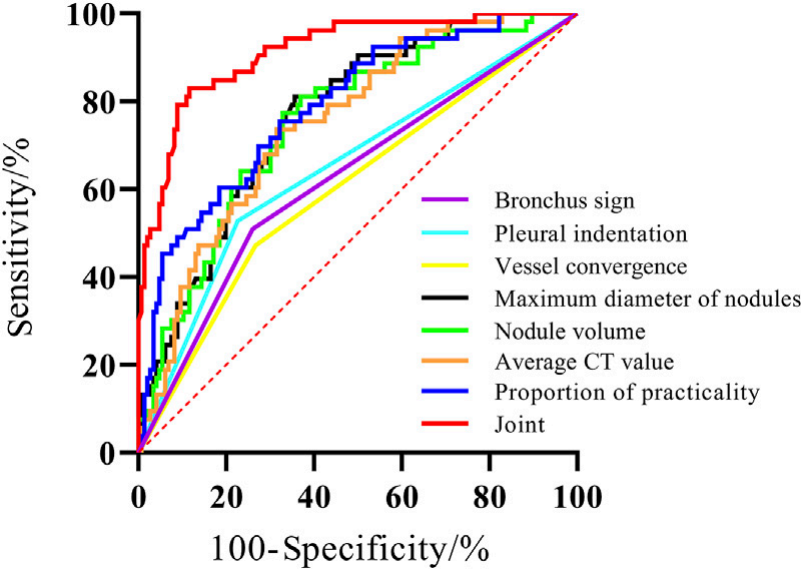
The ROC curve of three-dimensional CT qualitative and quantitative parameters on the prognosis of GGO-associated lung adenocarcinoma.

The AUC for BS, PIS, VBS, MND, NV, ACTV, and SCP were 0.625, 0.651, 0.602, 0.767, 0.755, 0.751, and 0.789, respectively. The AUC for the combination of these variables was 0.914, which was significantly greater than the AUC of the individual parameters (*P < 0.05*) (Figure 3; Table 6).

**TABLE 6:** The predictive value of qualitative and quantitative parameters of three-dimensional CT in the prognosis of GGO-associated lung adenocarcinoma.

Parameters	AUC	*95%CI*	Sensitivity (%)	Specificity (%)	Z	*P*
BS	0.625	0.553∼0.692	50.94	73.97	3.181	0.002
PIS	0.651	0.581∼0.717	52.83	77.40	3.903	0.001
VBS	0.602	0.531∼0.671	47.17	73.29	2.610	0.009
MND	0.767	0.702∼0.824	81.13	64.38	7.539	<0.001
NV	0.755	0.689∼0.813	77.36	67.12	6.752	<0.001
ACTV	0.751	0.685∼0.810	73.58	68.49	6.833	<0.001
SCP	0.789	0.725∼0.843	75.47	67.81	8.018	<0.001
united	0.914	0.866∼0.949	83.02	88.36		<0.001

## Discussion

Previous studies have reported a relationship between ground-glass opacity (GGO) and GGO-associated lung adenocarcinoma [9]. Compared to traditional two-dimensional computed tomography (CT), three-dimensional CT reconstructions based on artificial intelligence are more effective in diagnosing GGO. They can accurately localize the anatomical structure of lung nodules, visualize the density and marginal characteristics of pulmonary nodules, and significantly improve the detection rate of GGO, especially for small-diameter nodules [10].

Epidermal growth factor receptor (EGFR) and anaplastic lymphoma kinase (ALK) gene rearrangements are risk factors for the progression of lung adenocarcinoma. The EGFR and ALK genes are also the primary gene targets for targeted therapy in lung adenocarcinoma. Recently, many studies have explored the relationship between EGFR and ALK gene rearrangements and the imaging and pathological features of lung cancer, but there are many controversies [7, 11]. There is no clinical study on the relationship between three-dimensional CT imaging findings and EGFR and ALK gene rearrangements or prognosis in GGO-associated lung adenocarcinoma.

In this study, 41.83% of 208 patients with early-stage lung adenocarcinoma had EGFR gene rearrangements and 7.69% had ALK gene rearrangements. Meiying et al. [12] reported an EGFR mutation rate of 59.41% and an ALK rearrangement rate of 3.66%, which differ from our findings. These discrepancies can be attributed to several factors, including differences in sample size. Their study analyzed 547 patients with invasive lung adenocarcinoma, whereas our study included 208 patients with GGO-associated lung adenocarcinoma, encompassing both pre-invasive and invasive lesions. Moreover, differences in the maximum tumor diameter reflect variations in disease staging and inclusion criteria. Clinical characteristics of patients, such as age distribution, smoking status, and histological subtypes (e.g., acinar, papillary, and solid patterns), may also contribute to these discrepancies. Additionally, variations in genetic testing methods and sensitivity might have affected the detection rates of gene mutations. Furthermore, racial and regional factors, including genetic predisposition and environmental exposures, may also play a significant role in this discrepancy. Given the complexity of these influencing factors, further studies are needed to elucidate their effects on gene mutation and rearrangement rates, thereby enhancing our understanding of their clinical significance.

Further analysis of the qualitative and quantitative parameters of three-dimensional CT in patients with different EGFR and ALK gene rearrangements revealed that the BS, PIS, VBS, MND, NV, ACTV, and SCP were correlated with EGFR mutation in GGO-associated lung adenocarcinoma. These findings are consistent with those of Liu Y et al. [13]. Zhu Na et al^.^ [14] found that there were no significant differences in the MND, BS, PIS, and VBS between patients with EGFR mutation and those with wild-type EGFR. These discrepancies can be attributed to differences in the incidence of wild-type and mutant EGFR, smoking proportions, and histological subtypes. This study also found that the BS, PIS, VBS, ACTV, and SCP were correlated with the presence of ALK gene rearrangement in GGO-related lung adenocarcinoma, which is inconsistent with the findings of Han XY et al. [15]. The inconsistencies might be related to differences in the solid tumor volume of patients and the proportion of mucinous lung adenocarcinoma.

The EGFR gene, which encodes a transmembrane tyrosine kinase, is a commonly mutated gene in lung adenocarcinoma and is more frequently observed in elderly patients (≥60 years old), with a mutation rate of approximately 15%–50% [16]. The ALK gene is also commonly mutated in lung adenocarcinoma, with a mutation rate of approximately 3%–7%. ALK gene rearrangement is associated with tumor cell proliferation, differentiation, migration, neoangiogenesis, and apoptosis, making it an important target for treating lung adenocarcinoma [17]. Compared to EGFR mutations, which are more commonly observed in elderly patients, ALK rearrangements are predominantly found in younger patients (<60 years old), non-smokers, or light smokers with lung adenocarcinoma [6]. In malignant tumor cells, the pleural depression sign can stimulate tumor cells to release growth and signaling factors, promoting the proliferation and differentiation of fibroblasts in surrounding tissues and accelerating the growth of malignant tumors [18]. The VBS refers to the excessive proliferation of cancer cells in the lesion, resulting in irregular angiogenesis and cluster distribution, which is related to tumor growth, differentiation, and migration. It can also promote angiogenesis [19]. BS is related to tumors that surround the bronchial tubes but do not obstruct them. They are more pronounced when the fibrous fibers in the lesion shrink [20]. Compared to morphological characteristics, the quantitative parameters of three-dimensional CT can more accurately and efficiently determine the relationship between the CT characteristics of the lesions and gene rearrangements. Changes in the MND, NV, ACTV, and SCP are associated with tumor proliferation and differentiation, invasion, and vascular density [6, 21]. The progression of lung adenocarcinoma thickens the local lung interstitium inside the lesion, and cancer cells metastasize into the alveoli, thereby increasing tumor density, promoting tumor growth and metastasis, and accelerating tumor progression [22].

Several studies have confirmed that CT imaging findings are closely associated with the prognosis of GGO lung adenocarcinoma [23]. Wan GY et al. [24] found that the ACTV, SCP, and BS are predictors of disease-free survival in patients with lung adenocarcinoma. This study found significant differences in BS, PIS, VBS, MND, NV, ACTV, and SCP among patients with different prognoses.

In the study conducted by Wan GY et al., the maximum diameter of the lesion was ≤50 mm. The presence of satellite lesions, tiny calcifications, insufficient blood supply, increased formation of new blood vessels, rapid malignant proliferation, and a high degree of tumor malignancy were observed. ROC curves revealed that three-dimensional CT qualitative and quantitative parameters alone had poor predictive ability for the prognosis of GGO-associated lung adenocarcinoma, with low sensitivity or specificity. The AUC of the joint prediction model was 0.914, with the best sensitivity and specificity being 83.02% and 88.36%, respectively. This finding indicates that the combination of BS, PIS, VBS, MND, NV, ACTV, and SCP can predict the prognosis of GGO-associated lung adenocarcinoma, thereby helping to identify patients with a poor prognosis and improve their outcomes.

This study has several limitations. First, it focused solely on EGFR and ALK mutations without analyzing other potentially relevant driver genes, and the use of peripheral blood for detecting these genetic alterations may have increased false-negative rates. Second, the relatively short follow-up period restricted our ability to assess long-term prognosis and fully capture the evolutionary process of GGO-associated lung adenocarcinoma. Third, the single-center design and limited sample size may compromise the generalizability of the findings. Additionally, the study exclusively included patients with GGO-associated lung adenocarcinoma, excluding those without GGO, which limits the broader applicability of the results. Fourth, we focused solely on EGFR and ALK gene alterations and did not include other driver mutations. Although prior studies suggest a low K-ras mutation rate in GGO-dominant lung adenocarcinoma and mutual exclusivity between EGFR/ALK and K-ras alterations, future research should expand genetic profiling to assess the impact of multi-gene interactions on CT imaging features.

In summary, the quantitative and qualitative parameters of three-dimensional CT are related to EGFR and ALK gene rearrangements and prognosis in patients with GGO-associated lung adenocarcinoma. The combination of BS, PIS, VBS, MND, NV, ACTV, and SCP can help predict the prognosis of patients and formulate subsequent treatment plans.

## Data Availability

The raw data supporting the conclusions of this article will be made available by the authors, without undue reservation.
